# Controlling the Immune Suppressor: Transcription Factors and MicroRNAs Regulating CD73/NT5E

**DOI:** 10.3389/fimmu.2018.00813

**Published:** 2018-04-18

**Authors:** Theresa Kordaß, Wolfram Osen, Stefan B. Eichmüller

**Affiliations:** ^1^GMP & T Cell Therapy Unit, German Cancer Research Center (DKFZ), Heidelberg, Germany; ^2^Faculty of Biosciences, University Heidelberg, Heidelberg, Germany

**Keywords:** checkpoint molecule, CD73, NT5E, microRNA, transcription factor, T cell, tumor, A2A receptor

## Abstract

The NT5E (CD73) molecule represents an ecto-5′-nucleotidase expressed on the cell surface of various cell types. Hydrolyzing extracellular adenosine monophosphate into adenosine and inorganic phosphate, NT5E performs numerous homeostatic functions in healthy organs and tissues. Importantly, NT5E can act as inhibitory immune checkpoint molecule, since free adenosine generated by NT5E inhibits cellular immune responses, thereby promoting immune escape of tumor cells. MicroRNAs (miRNAs) are small non-coding RNA molecules regulating gene expression on posttranscriptional level through binding to mRNAs, resulting in translational repression or degradation of the targeted mRNA molecule. In tumor cells, miRNA expression patterns are often altered which in turn might affect NT5E surface expression and eventually influence the efficacy of antitumor immune responses. This review describes the diverse roles of NT5E, summarizes current knowledge about transcription factors controlling NT5E expression, and highlights the significance of miRNAs involved in the posttranscriptional regulation of NT5E expression.

## Functions of NT5E in Healthy Tissue and Tumors

**Figure 1:**
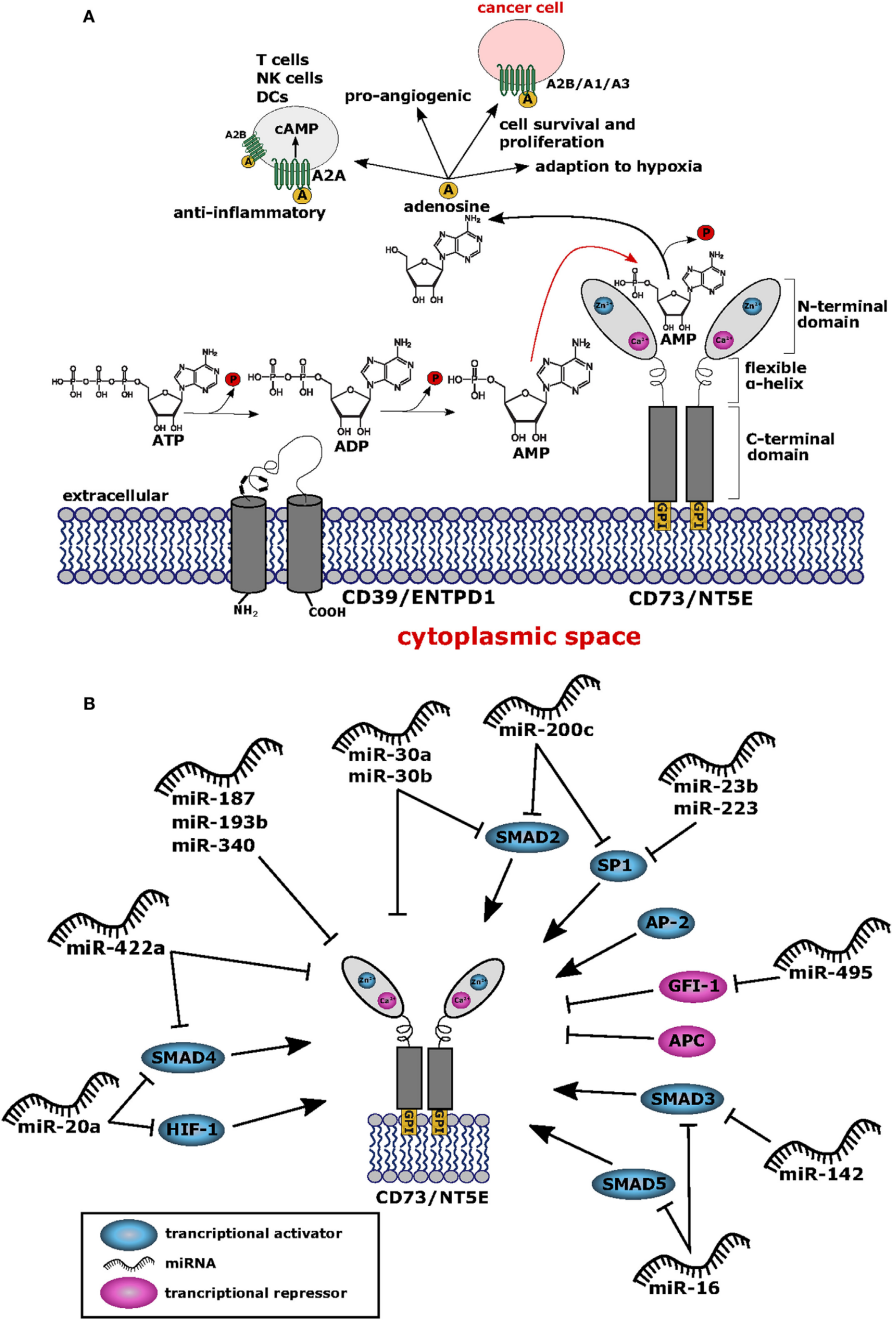
**(A)** Structure and function of CD73/NT5E. The membrane bound ecto-5′-nucleotidase NT5E hydrolyzes extracellular adenosine monophosphate (AMP) into adenosine and inorganic phosphate (P). Upstream of NT5E, adenosine triphosphate (ATP) is hydrolyzed *via* two reaction steps into AMP by the enzyme ectonucleoside triphosphate diphosphohydrolase-1 (ENTPD1) (CD39). Adenosine thus produced exerts anti-inflammatory effects by binding to the adenosine A2A receptor (ADORA2A) expressed by T cells, natural killer (NK) cells, and dendritic cells (DCs) resulting in cAMP mediated blocking of their effector functions. To some extent, the A2B receptor (ADORA2B) is also expressed on DCs and macrophages which are suppressed by adenosine. Thus, cancer cells can evade the immune system by upregulating NT5E protein levels. Furthermore, adenosine binds to the A2B receptor expressed by cancer cells leading to tumor cell survival and proliferation. Cancer cells also express the adenosine A1 receptor (ADORA1) and A3 receptor (ADORA3) and binding of adenosine to these receptors leads to tumor cell migration and proliferation *via* signaling through Gαi proteins. Adenosine is also involved in the adaption to hypoxia and shows pro-angiogenic potential. All adenosine receptors are depicted as stylized green transmembrane proteins. Adenosine is also symbolized as yellow circles marked with “A”. **(B)** Network of transcription factors and microRNAs (miRNAs) regulating NT5E expression. This network summarizes the current knowledge on regulation of NT5E on transcriptional (TFs) and posttranscriptional level by TFs and miRNAs, respectively. Transcriptional activators are depicted in blue and transcriptional repressors are highlighted in magenta. miRNAs targeting NT5E directly are shown, as well as miRNAs with indirect impact on NT5E expression through targeting of transcriptional regulators.

The membrane bound NT5E (CD73) is an ecto-5′-nucleotidase (NT5E) hydrolyzing extracellular adenosine monophosphate (AMP) into adenosine and inorganic phosphate (1) (Figure 1A). The enzyme consists of a homodimer inserted into the cellular membrane by glycophosphatidylinositol anchors. Besides hydrolyzing AMP to adenosine, NT5E has nucleosidase activity as shown for nicotinamide adenine dinucleotide and nicotinamide mononucleotide (2, 3). NT5E works in concert with ectonucleoside triphosphate diphosphohydrolase-1 (ENTPD1), which is also referred to as CD39, representing another ectonucleotidase acting upstream of NT5E catalyzing the hydrolysis of adenosine triphosphate (ATP) into AMP through two reversible reaction steps, whereas the final NT5E-mediated reaction from AMP to adenosine is largely irreversible (4) (Figure 1A). The two C-terminal domains of the NT5E molecule mediate noncovalent homodimer association and harbor the substrate binding sites (2). The molecular structure of NT5E can exhibit open or closed conformation and transition between these two stages occurs during substrate cleavage involving conformational changes enabled by the flexible α-helix connecting the C-terminal domains with the Zn^2+^ binding N-terminal domains (3), the latter being N-glycosylated at four distinct asparagine residues either by mannose saccharide chains or by a mixture of complex glycans and high mannose (2). Besides expression of the full-length molecule NT5EL (NT5E-201, 574 aa), a spliced version lacking exon 7 designated NT5ES (NT5E-203, 525 aa) can be detected in various human tissues and was found intracellularly overexpressed in human hepatocellular carcinoma cell lines (5). As depletion of amino acids 404–453 encoded by exon 7 prevents homo dimerization, NT5ES shows impaired substrate binding resulting in abrogated 5′-nucleotidase activity and lack of surface expression. Importantly, overexpression of NT5ES was shown to cause proteasome-mediated degradation of intracellular NT5EL, without affecting expression levels of native NT5EL dimers. Thus, altered splicing patterns commonly observed in many tumors (6–8) might contribute to aberrant NT5E expression levels in cancer cells.

Considering healthy tissue, expression of NT5E is detectable in epithelial cells of the respiratory tract, smooth muscle cells, and cardiac myocytes and other tissues, as can be deduced from the bioGPS mRNA expression data base (9) using the data set of Primary Cell Atlas (10). Under physiological conditions, NT5E has been described as a regulator of epithelial ion transport, thereby preserving mucosal hydration (11). Moreover, NT5E can act as gate keeper on endothelial cells as free adenosine facilitates “resealing” of gaps between vascular endothelial cells left behind by transmigrating neutrophils (12). Furthermore, adenosine generated through NT5E was described to restrict inflammatory immune responses through a negative feedback loop on adenosine receptor expressing neutrophils (13). NT5E has been found expressed on regulatory T cells (T_reg_) and at even higher levels on anergic CD4^+^ T cells, thereby preserving self tolerance in healthy individuals and protecting the fetus from maternal immune attack during pregnancy (14, 15).

Interestingly, qualitative differences in NT5E function have been described depending on the NT5E expressing cell type (16, 17). Comparing epithelial cells and lymphocytes, both expressing NT5E, lymphocyte NT5E was found susceptible to phosphatidylinositol phospholipase to greater extent compared to NT5E expressed by epithelial cells (16). In the same study, antibody binding to NT5E triggered shedding from the surface of lymphocytes, but not in the case of epithelial cells. A similar observation was reported by others who proposed NT5E shedding from the surface of B16F10 cells as explanation for absent cell surface staining on murine B16F10 melanoma cells despite detection of intracellular NT5E expression (18). The study by Airas et al. (16) demonstrated also signal transduction activity of NT5E expressed on lymphocytes, whereas NT5E expressing epithelial cells lacked this function. Signal transduction by NT5E appears unexpected since this molecule lacks intracellular signaling domains (Figure 1A); however, it has been suggested that NT5E might associate with src protein kinases, thereby facilitating cellular signal transduction as proposed by Wang and colleagues (19, 20). Alternatively, NT5E might also mediate signal transduction directly (19).

In pathophysiological situations, NT5E activity was found relevant for the generation of cardio-protective adenosine in the ischemic myocardium (21) or for adaption to hypoxia (see below). Importantly, NT5E is involved in tumor development. Thus, NT5E has been described to sustain tumor angiogenesis in murine tumor models of breast cancer and prostate cancer (20, 22) as well as in xenograft models of humans breast cancer (23). Likewise, NT5E expression promoted invasion and metastasis of murine and human melanoma cells (24) and of human breast cancer cells (25).

Notably, NT5E plays a significant role as immune-inhibitory checkpoint molecule (26). Thus, infiltration of tumors by NT5E expressing regulatory immune cells such as T_reg_ (27), MDSCs (28), or dendritic cells (DCs) (29) results in accumulation of immunosuppressive adenosine that can activate cAMP signaling in T cells expressing A2A adenosine receptors (ADORA2A). Moreover, adenosine receptors were found to be expressed on DCs, macrophages, MDSCs, and natural killer (NK) cells, implying that adenosine can repress the function of these immune cells (30, 31). Recently, an interesting phenomenon was described showing that T_reg_ undergoing apoptosis within the metabolically abnormal tumor microenvironment release substantial amounts of ATP, that is degraded by the nucleotidases of the faded T_reg_ resulting in accumulating adenosine levels (32). Adenosine can then trigger immune suppressive downstream effects among T cells like inhibition of chemotaxis, proliferation, activation, and effector function (33, 34). In light of its immune suppressive function and due to its expression by various tumor entities, such as melanoma (35–37), triple-negative breast cancer (34, 38), colorectal cancer (CRC) (39), and non-small cell lung cancer (40), NT5E has been considered as target checkpoint molecule for novel tumor immunotherapy approaches (41, 42). Indeed, injection of blocking NT5E-specific ab into tumor bearing mice resulted in reduced outgrowth of NT5E expressing tumors as shown for various tumor entities (43–45). Noteworthy, tumor cells can express adenosine A1 receptor (ADORA1) and ADORA3 receptors coupled to Gαi proteins, fostering tumor cell proliferation and migration (46). Moreover, therapeutic targeting of NT5E using specific inhibitors or blocking antibodies, respectively, has been proposed (22, 34, 38, 47–50) and is presently tested in a phase I clinical trial (NCT02503774).

In addition to its enzymatic function, NT5E can act as a receptor molecule shown to mediate cell–cell adhesion between lymphocytes and endothelial cells (51). Moreover, it was demonstrated that NT5E was can interact with extracellular matrix components (ECM) (34, 52, 53). This interaction occurred independently from enzymatic activity of NT5E, as blocking of ectonucleotidase function by concanavalin A did not affect interaction with ECM components like fibronectin, tenascin C, or collagen 1. In fact, NT5E turned out to mediate cell adhesion and migration *via* interaction with tenascin C (53).

Thus, NT5E appears to support tumor growth at multiple levels, i.e., by suppression of antitumoral immune responses *via* supply of adenosine and through facilitated dissemination of malignant cells from the primary tumor.

## Transcriptional Regulation of NT5E Expression

The promoter region of NT5E contains binding sites for the transcription factors SP1, AP-2, and SMAD proteins as well as cAMP-responsive elements (54, 55) (Figure 1B; Table 1). Chromatin immunoprecipitation showed that transcription factors SMAD2, SMAD3, SMAD4, SMAD5, and SP1 bind to the rat NT5E promoter, with SMAD5 and SP1 being most efficient (55). As rat and human NT5E transcripts share 89% identity (56), it appears possible that human NT5E expression might be regulated by SMAD transcription factors as well.

**Table 1:** List of transcription factors and miRNAs regulating NT5E.

Target	Regulator	Effect on NT5E	Host cell	Reference
NT5E	SP1	Activation	Human WI-L2	Hansen et al. (54)
			Rat hepatocytes	Fausther et al. (55)
NT5E	TFAP2A	Activation	Human WI-L2	Hansen et al. (54)
NT5E	SMAD2	Activation	Rat hepatocytes	Fausther et al. (55)
NT5E	SMAD3	Activation	Rat hepatocytes	Fausther et al. (55)
NT5E	SMAD4	Activation	Rat hepatocytes	Fausther et al. (55)
NT5E	SMAD5	Activation	Rat hepatocytes	Fausther et al. (55)
NT5E	HIF1A	Activation	Human T84 epithelial cells	Synnestvedt et al. (57)
			Human HepaRG cells	Tak et al. (58)
NT5E	TCF-1/β-catenin	Activation	Human HeLa and Jurkat cells, monkey Cos-7 cells	Spychala and Kitajewski (59)
NT5E	APC	Inhibition	Human SW480 colon cancer cells	Spychala and Kitajewski (59)
NT5E	NFκB/TNFα	Activation	Human HT29 colon cancer cells	Pagnotta et al. (60)
NT5E	PPARγ	Inhibition	Human HT29 colon cancer cells	Pagnotta et al. (60)
NT5E	GFI-1	Inhibition	Murine Th17 cells	Chalmin et al. (61)
NT5E	STAT3	Activation	Murine Th17 cells	Chalmin et al. (61)
NT5E	FOXP3	Activation	Murine T_reg_ cells	Zheng et al. (62)
NT5E	miR-422a	Inhibition	Human SCC61, SQ20B and HaCaT cells	Bonnin et al. (63)
NT5E	miR-30 family	Inhibition	Human colorectal cancer	Xie et al. (64)
			Human gallbladder cancer	Wang et al. (65)
NT5E	miR-340	Inhibition	Human gallbladder cancer	Wang et al. (65)
NT5E	miR-187	Inhibition	Human colon cancer SW480, RKO and SW620	Zhang et al. (66)
NT5E	miR-193b	Inhibition	Human pancreatic cancer	Ikeda et al. (67)
SP1	miR-23b	Inhibition	Human MM and WM tumor cells	Fulciniti et al. (68)
SP1	miR-223	Inhibition	Human gastric cancer MGC-803, SGC-7901 and BGC-823	Hu et al. (69)
SP1	miR-200c	Inhibition	Human gastric cancer MGC-803 and AGS	Tang et al. (70)
SMAD2	miR-200c	Inhibition	Human ATC-derived cells	Braun et al. (71)
SMAD2	miR-30 family	Inhibition	Human ATC-derived cells	Braun et al. (71)
SMAD3	miR-16	Inhibition	Human Osteosarcoma	Jones et al. (72)
SMAD3	miR-142	Inhibition	Human HT29 colon cancer cells	Chanda et al. (73)
			MDA-MB-231 breast cancer cells	Ma et al. (74)
SMAD4	miR-20a	Inhibition	Human HT29 and HCT116 colon cancer cells	Cheng et al. (75)
HIF1A	miR-20a	Inhibition	HeLa cells, primary human macrophages	Poitz et al. (76)
GFI1	miR-495	Activation	Human DAOY and D283 (medulloblastoma) cells	Wang et al. (77)
SMAD4	miR-422a	Inhibition	Human LHCN-M2 muscle cells	Paul et al. (78)

*MM, multiple myeloma; WM, Waldenstrom’s macroglobulinemia; ATC, anaplastic thyroid carcinoma; miRNAs, microRNAs; HIF-1, hypoxia-inducible factor-1; TCF-1, T cell factor 1; APC, adenomatous polyposis coli; T_reg_, regulatory T cells*.

Interestingly, hypoxia-inducible factor-1 (HIF-1) can directly bind to the NT5E promoter thereby activating NT5E expression (57), which is in line with the functional role described for NT5E in hypoxia adaptation (79). Thus, hypoxia resulting from uncontrolled tumor cell proliferation (80) might induce HIF mediated upregulation of NT5E expression on tumor cells. Another biochemical cascade often altered in tumors is the β-catenin-dependent Wnt signaling pathway (81). The promoter core sequence of NT5E is flanked upstream by a regulatory region containing consensus motifs for T cell factor 1 (TCF-1), representing a component of Wnt/β-catenin signaling pathway. In fact, expression of β-catenin could drastically enhance expression of NT5E. This upregulation was found to be dependent on the presence of TCF-1. Interestingly, the authors could also show that the antagonist of β-catenin, adenomatous polyposis coli protein, inhibits NT5E expression (59). The activation of NT5E expression by β-catenin was also confirmed by Pagnotta et al. who furthermore identified NFκB/TNFα as positive transcriptional regulators of NT5E (60). Seeking biomarkers for CRC, the authors applied a translational pathology approach and identified NT5E among others as a prognostic marker. In line with these findings, NT5E levels were found significantly upregulated in tumor specimens compared to normal colonic mucosa samples. Spranger et al. found that active β-catenin signaling was negatively associated with T cell infiltration in human melanoma samples. This was confirmed in authochtonous tumor models with inducible β-catenin expression, where absence of T cells was observed selectively in β-catenin expressing tumors (82). It is tempting to speculate that this immune suppressive effect on T cell infiltration might result from enhanced NT5E expression induced through β-catenin signaling. Of note, besides activating mechanisms on NT5E expression *via* NFκB/TNFα signaling pathways, also negative effects on NT5E expression through of PPARγ have been described (60).

In murine Th17 cells differentiated with IL-6 and TGF-β *in vitro*, IL-6 was found to activate Stat3, while TGF-β suppressed the transcription factor Gfi-1. As shown by the authors Stat3 sustained, whereas Gfi-1 repressed expression of ENTPD1 and NT5E through specific promoter binding, thus demonstrating transcriptional regulation of these exonucleotidases in Th17 cells through IL-6 and TGF-β (61).

A genome-wide analysis to identify forkhead box transcription factor (Foxp3) target genes in mouse led to the identification of Nt5e as one target gene of Foxp3 in mature T_reg_ cells (62). Foxp3 is a specific transcription factor expressed in murine and human T_reg_ cells and in recently activated human T cells (83). Thus, regulation of Nt5e by Foxp3 appears cell type specific and does not necessarily apply the same way to cancer cells. Noteworthy, high expression levels of FOXP3 in ovarian cancer has been identified as a prognostic marker for poor survival of patients (84).

## MicroRNAs (miRNAs) Regulating NT5E Expression

MicroRNAs are small non-coding RNA molecules that bind to the 3′ untranslated region (3′-UTR) of target mRNAs, thereby blocking translation or inducing degradation of the targeted mRNA molecule, respectively, depending on the degree of complementary among the interacting nucleotide sequences (85). In cancer cells, aberrant miRNA expression patterns resulting in impaired regulation of target mRNA expression is commonly observed. Tumor cell-derived miRNAs have therefore gained relevance as biomarkers and as prognostic factors as described (86–88). Of note, the 3′-UTR of NT5E comprises 1,774 nucleotides (89) (NM_001204813.1), exceeding the average size of a human 3′-UTR (90) approximately threefold. Thus, regulation of NT5E expression by miRNAs appears to be particularly restrictive. To date, only a few miRNAs have been described that directly regulate NT5E expression (Table 1). Bonnin and co-workers reported the regulation of NT5E by miR-422a in head and neck squamous cell carcinoma (HNSCC) patients. The authors found a significant negative correlation between expression levels of miR-422a levels and NT5E mRNA. Blocking of endogenous miR-422a by specific antagomiRs resulted in increased NT5E protein levels with enhanced enzymatic activity. Reduced levels of miR-422a correlated with shorter relapse free survival times in HNSCC, potentially due to overexpression of NT5E (63).

Recently, miR-30a was found to target NT5E in CRC (64). In this study, transfection with miR-30a reduced NT5E expression on the mRNA and on protein level and direct interaction of miR-30a with the NT5E 3′-UTR was demonstrated *via* luciferase reporter assays. Similarly, direct regulation of NT5E was described through miR-30a-5p in non-small cell lung cancer (91). Enhanced expression of NT5E was accompanied by reduced miR-30a-5p expression, whereas miR-30a-5p overexpression resulted in downregulated NT5E expression on mRNA and protein levels. At the same time, proliferation, cell migration, and invasion of these cells were significantly reduced. These effects were mimicked by silencing NT5E expression using shRNA directed against NT5E.

Interestingly, the miR-30 family shares the same seed sequence (92). Thus, other miRNAs from this family might also regulate NT5E. Indeed, direct regulation of NT5E by miR-30b was shown by Wang et al. in gall bladder carcinoma (GBC). Including miR-340 in their study, the authors found that overexpression of miR-30b or miR-340 reduced GBC cell proliferation, migration, and invasion. For both miRNAs, direct interaction with the NT5E 3′-UTR could be verified and NT5E overexpression partially reverted these miRNA-mediated effects in GBC cells (65). In CRC, miR-187 levels were found strongly downregulated compared to adjacent normal tissue leading to the establishment of miR-187 expression levels as prognostic marker for CRC patients. In fact, transfection of miR-187 reduced cell proliferation and migration *in vitro* and decelerated tumor growth of CRC lines *in vivo*. In the same study, direct targeting of NT5E by miR-187 was demonstrated (66). Studies focused on miRNAs involved in the MAPK pathway of human pancreatic cancer cell lines revealed miR-193b as a direct binder of the NT5E 3′-UTR. However, in this study, binding specificity using a mutated reporter plasmid was not controlled and effects of miR-193 overexpression on NT5E expression on mRNA and protein level were not analyzed (67).

Considering the extraordinary size of the NT5E 3′-UTR region, the restricted number of validated miRNAs identified so far that directly target NT5E mRNA most likely represent just the tip of the iceberg. Further studies are needed to broaden the spectrum of known miRNAs that directly regulate NT5E surface expression, thereby potentially affecting the tumor cells’ vulnerability toward immune attack.

On the other hand, miRNAs can also function *via* indirect circuits, for example, by targeting transcription factors of NT5E, opening an alternative route for miRNA-mediated regulation of NT5E expression. In fact, miR-23b was found to directly suppress expression of transcription factor SP1 in multiple myeloma cells (68), and in gastric cancer, an inhibiting effect of miR-223 on epithelial to mesenchymal transition *via* direct posttranscriptional silencing of SP1 was reported (69). Other authors described miR-200b and miR-200c as direct inhibitors of SP1 transcription within this tumor entity (70). Whether the miRNA-mediated inhibition of SP1 expression resulted also in downstream reduction of NT5E expression levels was not investigated in these studies.

In a study by Braun et al., the authors focused on the identification of miRNAs affecting the invasive potential of anaplastic thyroid carcinoma and found miR-200c and miR-30a–e to target SMAD2 (71), representing another transcriptional activator of NT5E. Similarly, miR-16 might indirectly downregulate NT5E expression, as this miRNA was shown to inhibit expression of the transcription factors SMAD3 and, to lesser extent, SMAD5 in human osteosarcoma lines (72). Furthermore, SMAD3 is also targeted by miR-142-5p as shown in human rotavirus infected cells as well as in human breast and lung cancer cell lines (73, 74).

Further examples of miRNA-targeted transcription factors of NT5E are SMAD4 and HIF1A downregulated by miR-20a-5p (75), and Poitz et al. showed the direct downregulation of HIF1A by miR-20a (76). As mentioned above, miR-422a was shown to directly regulate NT5E, however, SMAD4 has also been described as a direct target of miR-422a (78), suggesting that miR-422a has the capacity to decrease NT5E levels both directly and indirectly. One example for a miRNA that could indirectly lead to an upregulation of NT5E levels is miR-495, which was shown to target one of NT5E’s transcriptional repressor GFI1 in medulloblastoma cells (77).

## Conclusion

NT5E (CD73) has emerged as a novel target for tumor immunotherapy approaches, since functional inhibition of NT5E reversed its immunosuppressive effects resulting in tumor immune attack and eradication of cancer cells by cytotoxic CD8^+^ T cells and NK cells. Knowledge about NT5E regulation on the transcriptional and posttranscriptional level might provide a deeper understanding how cancer cells acquire aberrant NT5E expression to facilitate immune escape. We suggest a complex regulatory network of activatory and inhibitory transcription factors acting in conjunction with miRNAs to control NT5E expression. Interestingly, certain regulators such as miR-422a exert their effect on NT5E expression both directly as well as indirectly, i.e., through binding to the 3′-UTRs of NT5E mRNA and SMAD4 mRNA, the latter representing a transcriptional activator of NT5E. Even though the 3′-UTR region of NT5E is of extraordinary size, only few miRNAs have been described so far that regulate NT5E expression. Identification of further miRNAs targeting NT5E will help to unravel the complex regulation of NT5E expression in cancer cells.

## Author Contributions

TK, WO, and SE wrote this paper. TK generated the figures.

## Conflict of Interest Statement

The authors declare that the research was conducted in the absence of any commercial or financial relationships that could be construed as a potential conflict of interest. The reviewer IS and handling Editor declared their shared affiliation.
